# Personalising clinical pathways in a London breast cancer service

**DOI:** 10.1111/1467-9566.13441

**Published:** 2022-02-10

**Authors:** William Viney, Sophie Day, Jane Bruton, Kelly Gleason, Charlotte Ion, Saima Nazir, Helen Ward

**Affiliations:** ^1^ Department of Anthropology Goldsmiths, University of London London UK; ^2^ 4615 Patient Experience Research Centre School of Public Health Imperial College London London UK; ^3^ Cancer Research UK Imperial Centre Faculty of Medicine, Imperial College London London UK; ^4^ Breast Cancer Translational Research Imperial College London London UK; ^5^ 129367 Breast Cancer Services Charing Cross Hospital London UK

**Keywords:** breast cancer, clinical pathways, personalisation, personalised medicine, translational research

## Abstract

Using interview and observational data from a busy and research‐intensive breast cancer service in the United Kingdom, we discuss recent developments in personalised medicine. Specifically, we show how clinical and research practices meet in clinical pathways that are reconfigured in response to changing approaches of diagnosing, monitoring, treating and understanding cancers. Clinical pathways are increasingly sensitive to changes in evidence deduced through new technologies and therapies as well as decisions based on intensive, iterative analysis of data collected across a range of platforms. We contribute to existing research by showing how the organisation of clinical pathways both maintains established clinical practices and responds to new research evidence, managing a threshold between evidence‐based and experimental medicine. Finally, we invite comparisons with other forms of personalisation to understand how they depend on the ‘real time’ collection, analysis and application of data.

## INTRODUCTION

Translational priorities to conduct research that can be applied rapidly in clinical settings have led to the creation of more targeted treatments, seen by many as a key component of ‘personalised’ or ‘precision’ medicine in the United Kingdom and internationally (see Cambrosio et al., 2018; Day et al., 2021; Hedgecoe, 2006; Keating & Cambrosio, 2003, 2011; Tutton, 2012). Commentaries in social and clinical sciences have also highlighted the intensity, speed, and scale of investment in research and development, including the synthesis of clinical, behavioural, and high‐throughput genomic sequencing data (Cambrosio et al., 2018; Kerr et al., 2021; Leonelli & Tempini, 2020; Richardson & Stevens, 2015; Stevens, 2013; Tempini & Leonelli, 2021). In combination with pharmacogenomics, precision surgery and radiotherapy, these practices are expected to converge on fast, large throughput of data that can adjust the understanding of a patient's diagnostic and prognostic status in what is called ‘real time’ (see Kerr et al., 2021, p. 166). Such commentaries conclude that ‘oncology has become a significant extent to a data‐centric domain’ (Bourret et al., 2021; see also Hoeyer, 2019).

Through ethnographic engagements in a National Health Service (NHS) hospital, we document the everyday work of ‘personalising’ medicine and research in breast cancer care. We focus on the day‐to‐day organisation of clinical pathways, which implement personalised medical innovations. These pathways are tools that plot steps through treatment for a particular health condition, guiding decisions at the local level in accordance with patient preferences and the assessments of multidisciplinary teams (Martin et al., 2017; Pinder et al., 2005). Also known as ‘integrated care pathways’, ‘critical pathways’, ‘care plans’, ‘care paths’ or ‘care maps’ (see Rotter et al., 2010, p. 2–3), they encode NHS guidelines and monitor costs, rationing, workflow and performance management as well as organising patient ‘journeys’. A Cochrane review summarised that ‘clinical pathways are document‐based tools that provide a link between the best available evidence and clinical practice. They provide recommendations, processes and timeframes for the management of specific medical conditions or interventions’ (Rotter et al., 2010). In the field of oncology, thanks in part to the developments in translational research, clinical evidence is changing rapidly and provide provisional and mutable guides to care, since they respond to changing disease categories and treatments, and techniques of monitoring and management.

Social scientists have shown that the maintenance of ‘standard’ clinical pathways involves considerable variation and adaptation through the data systems, processes and people who combine to ‘torque’ patient and occupational biographies according to encounters with protocol: they involve constant innovation to achieve ‘local universality’ (Timmermans & Berg, 1997). Established clinical pathways are by no means simple or straightforward. They require significant technical and other resources to establish and maintain, and, like other infrastructures of classification, produce numerous known and unknown consequences (Bowker & Star, 1999). Vinh‐Kim Nguyen showed how triage into different clinical pathways was an unintended and unanticipated by‐product of ‘improvising medicine’ (Livingston, 2012) as much as an intended stratification (Nguyen, 2010). In this sense, clinical pathways respond to, anticipate and produce change, albeit at rates that vary widely according to circumstance. We aim to contribute to this research by describing the routine work of orchestrating pathways that may not be predictable and cannot be comprehensively known ahead of time.

Once treated as effectively a single disease, breast cancer now consists of four molecular subtypes and at least 21 distinct histological subtypes (referring to where cancerous tissue originates) that differ in risk factors, presentation, response to treatment and outcomes (American Cancer Society, 2020; National Institute for Health and Care Excellence, 2018). Yet, these subtypes are changing in response to emerging information about biological markers, which are combined or ‘layered’ (Ross et al., 2021) with existing disease subtypes, treatments and methods for monitoring. We observed how clinical pathways have become more flexible, as understandings of diagnostic and treatment methodologies have evolved with trials and other studies used to monitor developments.

The ethnographic descriptions central to this article show staff ‘supporting’, ‘following’ and ‘extending’ clinical pathways to support patient care and research. These perspectival renderings capture how health care dynamically stratifies patients into evolving cancer types and differentiated journeys, according to changes in research evidence, service structure and resources, as well as patient‐level changes that may also combine diverse health conditions and comorbidities. Our focus on the daily work of coordinating these pathways shows that the combination of new with existing elements of pathways produces a sense of differential rates, rhythms and tempos of action and expectation (Day et al., 2021; Feiler et al., 2017; Kenny et al., 2021; Tutton, 2012). While it is to be expected that the combination of ‘new’ and ‘old’ methods of diagnosing, monitoring and treating disease as well as pacing and costing the associated work will have unexpected as well as predicted results, we also aim to contribute to the existing literature by asking what might count as the ‘right’ or ‘real time’ tempo, or timeliness, of medical and other personalising practices. Staff and patients in this NHS service navigated adaptive clinical pathways, combining standard and experimental practices in uncomplicated, early treatment, as much as ‘time‐taming’ end‐of‐life care and research (Bogicevic & Svendsen, 2021). These adaptations affected workloads and job tasks for staff, logistics, patient hopes, requirements for new testing procedures, and the interactions and tensions between research and clinical care. At the end of this article, these themes guide our group's concluding remarks on the wider contexts of personalisation within and beyond medicine.

## METHODS

We make visible the work of managing and adapting clinical breast cancer pathways in relation to service developments, new technologies and biomedical research by providing accounts of the work of a clinical pharmacist, a research coordinator and an oncologist. These three descriptions—shown from the perspective of one of our group (W.V.)—show how clinical pathways are interwoven with research activities, and how these merge and diverge at different points, with important consequences for staff and patients in the service. Each member of staff was purposively selected because of their critical role in maintaining and extending clinical pathways. Personalised or precision oncology requires an increasingly granular approach to breast cancers, and these descriptions, drawn from informal conversation, semistructured interview and observation with staff and patients, show how evidence is translated along cancer pathways through close but not always smooth coordination of care and research.

Previously, we reported our findings from 2013–2014 in this London setting (Day et al., 2016), and we used earlier fieldwork as a guide to our work from 2018–2020. Our methods mirrored the combination of observation and interview of our previous study, but we aimed to document how the service had changed. We wanted also to explore the continuing effects of iteratively implementing translational research on staff and patients. Over an 18‐month period, we observed the main outpatient clinic and accompanied staff and patients to other parts of the clinical pathway, such as chemotherapy services or multidisciplinary team meetings. We also interviewed 26 members of staff and 28 patients, including 4 staff members and 7 patients who had participated in the previous study. Initial interviews were semistructured and aimed to elicit relevant histories of illness and occupation. In staff interviews, we focussed on occupational themes, changes in service structure, workloads, research practices and findings, clinical, technological, and organisational practices and approaches. In patient interviews, we focussed on experiences of care and research; some participants had recently been diagnosed with cancer, but others had interacted with the service for decades and were interested in describing changes in the service. In all interviews, we elicited accounts about personalising practices such as targeted treatments, adaptions to the care pathway and participation in research studies. Key themes were informed by previous research in this service, and emerging themes were identified and discussed in monthly team meetings before coding, enabling a shared foundation for continuing fieldwork. Observations allowed us to position participants in clinical pathways and to further contextualise interview material. Two researchers (W.V. and S.D.) carried out observation and interviews and kept field notes. Interviews were recorded and transcribed verbatim, coded and analysed in NVivo12. The integration of these varied data enabled fuller interpretation: interview material was extended with observations of participants across different settings, while fieldwork notes were elaborated in the light of individual and collective views and explanations (see Day et al., 2016, 2021).

## SUPPORTING PERSONALISING CLINICAL PATHWAYS

The National Institute for Health and Care Excellence (NICE) Pathways provide guidance in interactive flowcharts for early and locally advanced, familial and advanced breast cancer.^1^ These national pathways interlink the sequence of scanning, investigation, intervention, monitoring and management support with the best available clinical evidence. While *a priori* mapping of clinical pathways meets the challenge of standardising protocols, they must also anticipate emergent properties of newly recognised ‘diseases’ or ‘disease markers’, individual disease progression and treatment response, which cannot be known ahead of time. Moreover, and in a translational environment fostering personalised approaches to diagnosis and treatment, clinical pathways are fundamental to the organisation of research.

At the time of our more recent observations, approximately 700 patients were newly diagnosed in the breast service each year, a tertiary referral centre for West London. Prior to the SARS‐CoV‐2 pandemic, the service screening hub saw as many as 60,000 asymptomatic people a year for breast cancer. Following investigation and diagnosis, the centre provides surgery, radiotherapy, oral or intravenous chemotherapy, and a variety of other treatment and support services. Different treatment modalities mean that patients are seen and supported by different clinicians and teams at different ‘stations’ along a pathway of care. For example, cancer nurse specialists (CNS) work throughout the pathway, collaborating closely with oncologists, pharmacists, administrators and others. We attended weekly multidisciplinary team meetings (MDTs) where primary treatment plans for individual patients were discussed and clinical decisions made.

Routinely collected data on patient visits, tests, diagnoses and treatments combine with tissue, drug, observational and other studies embedded into different parts of clinical pathways via local translational partnerships. For instance, the hospital contributes to one of the UK’s Academic Health Science Centres, links to busy clinical trials units and networks, translational research institutes and shared platforms that bring private industry, academics and clinicians, funders, investors and regulators together. The service facilitates studies in translational genomics, metabolic phenotyping, imaging technologies and health informatics, using administrative and clinical data from a recently established data warehouse, research data and samples stored in institutional biobanks.

Previous work (Day et al., 2016) observed a service adjusting to a merger of hospital group services into a single hospital site. According to staff, we interviewed, the service had stabilised and was better staffed since our previous study; it was able to adapt, respond to and support changes in care and treatment. We found members of staff using new methods to coordinate changing clinical pathways in response to new kinds of treatment and data that track patient response:

At the centre of the hospital’s main oncology outpatient’s department (OPD) was ‘The Hub’—two small rooms and many computers, the walls dominated by large electronic screens displaying patient lists for the day’s clinics. Had I visited the outpatient’s department five years previously then I would not have entered the Hub or met Catherine^2^. The Hub was created as part of a co‐design project in 2014–2015 led by the Chief of Service/Trust Cancer Lead and supported by members of our research group. I first met Catherine in 2019 and we frequently interacted while I observed staff and patients. She was the lead clinical pharmacist in the oncology outpatients department and her role was created in 2015 to respond to new anti‐cancer treatments introduced into the service. She issues, manages, and monitors prescriptions electronically, provides in‐person support to consultants and registrars, as well as seeing patients of her own.

Clinical appointments were pre‐booked in this OPD and were coordinated by the tumour group. They ran for approximately 4 hours. Catherine was always present in the OPD. One change brought in as part of the 2014–2015 redesign was the pre‐clinic ‘huddle’ where doctors and other healthcare staff prepare by discussing the needs of individuals, dividing patients and duties between them. These are further opportunities to review, discuss and adjust patient’s place within a pathway of clinical care. Catherine kept to her corner, largely uninvolved in these preliminary deliberations, but her work changed as the doctors started to see patients. Doctors returned from their consultation rooms to make quick checks with her—‘Cath, are there fortnightly bloods for …?’ or ‘Do I do an ECG when starting …?’—and they got an answer and returned to their patients. She remained in the Hub to take these questions about eligibility, monitoring, trials, licencing and side effects, and through separate databases tracked and administered the funding status of different treatments.

When we met I felt as if I had joined a long queue of people who wanted to ask Catherine questions. I told her she was a professional information service and she laughed, explaining how the growing volume and variety of oral chemotherapies and targeted treatments meant there had been dramatic changes in how patients were managed. When she started in oncology pharmacy around fifteen years ago, she said, screening for chemotherapy was relatively straightforward. ‘Now there’s practically a new drug every week’, and she laughed again. Training new oncology pharmacists had become more challenging; there was simply so much to learn. In addition to licensed treatments she had to be aware of the protocols of drug trials to support trials coordinators. When we met my eye was drawn to a spreadsheet pinned to the wall by her desk. The chart detailed oral chemotherapies across all tumour groups, 36 treatments in total, and each treatment had separate monitoring requirements such as glucose, lipids, biomarkers, weight, temperature, electro‐cardiogram and more—a list that spread across pages, with a section for specific requirements, such as the timings of blood monitoring or particular adverse outcomes to observe. Testimony to the speed at which changes were being introduced, the sheet was annotated with treatments added since it was printed.

Improvements in treatments meant that some patients could partially self‐manage their chemotherapy. Catherine described this as ‘enablement’—allowing greater patient independence. While some tasks have moved from staff to patient other tasks were shifted, minimised, or enlarged across different parts of the hospital. Oral chemotherapy and targeted treatments such as CDK4/6 inhibitors, created additional work throughout the hospital system. Each new treatment had to be manually built into ARIA, the electronic prescribing system used in the Trust since 2011, trialled and monitored with patients before being used and routinely updated. Although Catherine thought developments enabled patients to manage aspects of their treatment, she also highlighted restrictions on physical space: capacity in phlebotomy and haematology had not kept pace with new treatment regimens. Patients returned to the hospital frequently and could wait many hours for prescriptions to be issued. She explained that prescribing oral chemotherapy treatment required patients to come early in the morning for baseline bloods, wait for the results to be reported so that the prescription can be raised and then sent to the pharmacy. This ought to be a 1‐day service, she said, but the pressure on phlebotomy and haematology grew ever greater.

Catherine met patients already diagnosed, sorted and matched to breast cancer's growing number of subtypes and treatment plans. She managed their access to licenced and off‐license treatments and participated in further developments to support clinical trials. Meanwhile, it was hoped that data fed back from patients on licenced treatments and trials to contribute to a growing evidence base informing further licencing, review and renewal decisions made by NICE. One generalised effect of this logic of adaptation is broadly temporal—the breast cancer service and its practices are placed in an expectant state of intense, condensed and dynamic change.

She mentioned three drug therapies that were introduced into the service between 2017 and 2019—palbociclib, ribociclib and abemaciclib—whose positive clinical outcomes were accompanied by changes in workloads and logistics.^3^ In breast cancer care, they are used in combination with existing drugs used to inhibit oestrogen production such as letrozole, anastrozole or exemestane, initially in patients with advanced metastatic disease. These new treatments illustrate a fast‐moving environment in which cancer types are mapped to potential treatments that change over time. As tumours develop resistance to therapies, patients may also become eligible for a range of research studies. In the case of CDK4/6 inhibitors, approximately 50 patients were being treated with them in the service when we were there, but the monitoring they required added approximately 700 annual appointments when bloods were taken, haematology ordered, reported and reviewed. At the threshold of research and the mainstream adoption of these inhibitors into clinical use are people like Catherine, who supported and maintained treatment protocols, helping to ensure NICE guidance is implemented through the service's clinical pathways, and data are collected to test and develop evidence of their effectiveness.

When we asked healthcare staff if they referred to pathways or used other maps or tools to help guide their decisions, they replied that they were more likely to make decisions in multidisciplinary team meetings, adapting and adjusting plans based on different forms of expertise. Service delivery interacts with and responds to developments in disease categorisation and treatment, mediated by innumerable kinds of feedback. Emerging evidence about treatments for different types of breast cancer led a senior oncologist to comment about their rate of introduction: new treatments, he said, were being introduced ‘at a much more rapid rate than the National Health Service can absorb the complexity. …[T]he machinery in the National Health Service is so cumbersome that it has trouble keeping up with the new diagnostic and new treatment methodologies that we have’. Moreover, he concluded, each patient has a unique cancer:

we’re in this era where it’s expanding the varieties and number and types of cancer due to the molecular characterisation of each patient’s cancer … virtually every patient has a different type of cancer due to the genetic changes that occur in the cancer compared with normal tissue.

Definitions of personalised or precision medicine differ in emphasis but agree on the importance of a ‘learning healthcare system’ with inbuilt characteristics of continuous optimisation and calibration. This logic depends on serial testing and retesting, especially in disease conditions that are ill‐understood (see Day et al., 2021). For example, NICE now recommend retesting hormone receptor status when services detect metastases, in addition to regular monitoring. Changes in a cancer, responses to treatment and other factors inform timely developments in the cancer pathway such as a change in treatment or eligibility for a research study. More generally, a clinical pathway emerges as a situation comes to be known in practice rather than in advance. It is not a set of ‘care processes for a well‐defined group of patients during a well‐defined period’ (Schrijvers et al., 2012, p. 1). To the contrary, what we call ‘personalising pathways’ emerge through sorting patients into categories and testing if they fit with their category over time, based on response to treatment and monitoring of disease. This dynamic stratification is neither reflected in ‘standardised’ mappings, nor does it always follow predetermined strata, particularly for advanced or metastatic disease.

## FOLLOWING PERSONALISING PATHWAYS

During our fieldwork, we worked alongside a group of research coordinators. They tended to be educated to undergraduate or graduate level biomedical sciences and provided on‐site training in project management, phlebotomy and sample preparation under the supervision of an experienced research manager. The team's primary role was to collect samples and study data for a range of observational and interventional research studies, many of them ‘basic’ in terms of their direct clinical impact,^4^ and yet the intersection of basic or experimental research and clinical care required constant management. The translational research activities supported by this group sought to improve the integration of research into clinical pathways. The work of following personalising pathways was shown to us by one research coordinator:

I followed Susie up and down the corridors of the hospital, through the many heavy double doors that link hospital departments. Susie’s pace was brisk as she talked to me over her shoulder. I doubled my step to keep up. She was wearing green scrubs, polymer clogs, and her hair tied up, making her indistinguishable from a member of clinical staff. As a research coordinator and part of the hospital group’s centre for experimental cancer medicine, her primary role was to set up collections and collect tissue samples for basic scientific research through the local tissue bank. On this occasion she was collecting tissue to send to the laboratory, with the hope that it would contribute, along with other samples, to a better understanding of cancers.

There were times when the work of care and research merged and complemented each other, and other times when these activities were held separate or in tension. Susie and other research coordinators struggled to follow unexpected changes in appointment times and treatment plans; they did not always know which patients were eligible for one or other of the studies in their portfolio. And so Susie needed to identify colleagues to work with her and let her know of changes in clinical schedules. She worked hard to build and maintain links between clinical pathways and research practices that underpinned this translational system.

According to national regulations, samples can only be taken as part of an existing elective procedure and so patients can only be consented for research in the context of the clinical pathway, for example, when bloods are taken or an operation scheduled. This means that these research materials have to be collected through clinical pathways, as they are enacted within the contingent conditions of hospital care. If the time changes for elective procedures, if a department is running late or has changed its schedule, if a surgical theatre has been delayed or a surgeon has brought a patient forward, then research coordinators must shadow these developments.

We waited as the patient arrived in interventional radiology and introduced ourselves to the clinician leading the biopsy. The healthcare worker told us they’d be delighted to take the sample and agreed that Susie can approach this patient. We waited, admiring the machine that performs the biopsy and passed the time with other staff, waiting in scrubs, making hot drinks and chatting. After a while the patient arrived and we said hello as the clinical team started a series of tests and asked lots of questions, checking she was ready for the biopsy. We waited out of sight for the right moment to approach so Susie could invite them to be in the study. The moment came: ‘Hi, I’m Susie from research. I’m wondering if you would be willing to give extra tissue for a project we are…’ ‘No’ replied the patient with a firm and friendly smile, ‘one is enough, thank you’. The exchange, hurried and impersonal, was over in a minute. We thanked them and retreated. After an hour of screening, waiting, preparation—it was over. I expected the patient to want to know about the research but Susie shrugged; things do not always go to plan, care comes before research.

As Susie and I returned to an office in the medical oncology department, we were met by her colleagues who were sifting through clinical and research data, looking for patients eligible for studies. Data systems come in a number of forms. Since patient data have been digitalised and shared within the Trust, research coordinators accessed patient records to screen patients for studies and frequently referred to clinical letters used by clinicians for summaries of staging and histology, surgery and treatment. On one wall of the office was a colour‐coded whiteboard. At the end of each week, Susie's manager gathered the group around this weekly planner—separated into morning and afternoon clinics with the names of senior oncologists whose clinics run on different days. On it were the week's tallies—12 blood samples for one study, 5 tumour samples for another—samples to be used in basic laboratory studies that rely on tissue bank infrastructures for storage, use and reuse in a range of studies. During these weekly planning and review meetings, they would congratulate and commiserate one another before these numbers. I learned that some samples could be easier to get than others, some types of sample were rare and others more inaccessible. The clinical circumstances for consent and collection make blood samples from women who were being diagnosed in the breast cancer service more numerous, but the samples were collected at times of great emotional stress. Some grades of tumour or samples from patients who have not undergone systemic treatment had particular value. Susie needed to know when patients have departed standard treatments, as it affects their eligibility for the studies she manages, ‘we don't always know where a patient's going to be at various points of their pathway because they don't always follow the same path through their treatment’. The more treatment pathways changed the harder it was to integrate sample collection and clinical schedules.

Personalising pathways are driven by the rapid application of research findings, and so, conditions for conducting research are subsequently transformed. Sonia, a patient we met during her treatment was asked by a research coordinator to participate in basic translational research through donation to the tissue bank. Her diagnosis was not straightforward and she needed multiple biopsies. She participated in research following tests to confirm the hormone receptor status of her tumour. She recalled the sudden and abrupt way her surgeries were organised to halt the growth of a primary tumour she was told was especially aggressive, with a 95% chance of recurrence. After radiotherapy and chemotherapy, other women Sonia had befriended in the clinic finished their treatment, and their WhatsApp group became a place to discuss post‐treatment experiences and follow‐up care. Meanwhile, her chemotherapy continued: she had a strong adverse reaction to a trial treatment leading to a short time in inpatient care; her chemotherapies were changed, doses adapted and changed again to work in combination with pertuzumab (Perjeta) and trastuzumab (Herceptin). She reported a sense of bewilderment about the organisation of her treatment and her own status—‘I don't know what category I am. I have no idea’. She felt that her needs were conflated with those of her tumour; her care optimised around this priority in such a way that her own knowledge and understanding were held in suspended animation, punctuated by testing and retesting: ‘so many tests but no real information, no feedback about [what] this is going to do, this is the result of this test or this is going to be targeted, whatever, to the next’. The steady accumulation of data helping to shape individual patient journeys meant patients like Sonia became aware of the various factors that influenced how her pathway was being formed—her relatively young age, premenopausal status and underlying health conditions. But she felt that tumour classification, testing and monitoring of response, was the principal influence that shaped her interactions with the service. They also informed her participation in research: she became eligible for a drug trial in which she briefly participated and donated tissue samples to researchers during clinical investigations. Known in retrospect, changes to her clinical pathway were shaped by the treatment she had already and the opportunities available in the hospital at the time (Nguyen, 2010).

Patients used varied analogies and words to describe the duration and tempo of their care and research participation, and we were led to view these combined activities as shaping the chronology and topography of personalising clinical pathways. In a group discussion with patients and staff from different cancer services, one patient likened their diagnostic pathway to being on a conveyor belt and part of a factory system, their treatment mass produced and their part played as in a script. Metaphors such as these suggest a rapid and uniform system at first. With time, their experiences settled, broadened and the pace of their interactions stabilised. Patients we interviewed did not use the term ‘clinical pathway’ to describe their care, but they often referred to their ‘journeys’. While some journeys were brisk, following a common clinical pathway from diagnosis to discharge, others proved long and unpredictable, particularly when initial diagnostic tests were inconclusive. Many also said they were confused when their treatment changed, and their journey took a new direction. The direction of their ‘pathway’ was not always transparent to them or indeed anyone. It was unclear who or what was driving changes in their assessment, and frequent monitoring could cause them to wonder if they were in the ‘right’ category and whether their tumour had evolved.

Nurses were likened to a ‘SatNav’—according to one manager—that guided patients through the service from diagnosis to treatment and follow‐up. But outcomes from newer drug combinations made it hard to determine the likely sequence of steps. One nurse explained that while new treatments like CDK4/6 inhibitors extend the lives of some patients, ‘the long‐term data just isn't there, because they've only been used in trials for so many years’. She referred to developments leading monoclonal antibody treatments like trastuzumab (Herceptin), commonly credited as a ‘poster child’ for personalised medicine (Hedgecoe, 2004; MacMahon, 2020; Reynolds et al., 2014) to be combined with or supplanted by newer therapies, as they were for Sonia. This nurse described patients currently treated with combination antibody treatments, trastuzumab and pertuzumab, with surprising results. She knows one case, a young patient with metastatic cancer who no longer has measurable disease, something this nurse had not seen before. She wondered: ‘how do we know that she's still got metastatic breast cancer when she has no cancer that we can measure or see?’ This patient no longer belonged to the pathway into which her metastatic cancer once placed her—she had entered a new, potentially unique pathway and staff supporting her were required to improvise. ‘This is new; it's unchartered territory’. Such cases were a source of great hope, excitement and uncertainty. We asked if a clinical pathway influenced how this nurse supported patients in these circumstances. She said she rarely referred to a mapped protocol or decision tree, the service was too busy and clinical pathways did not include the drug trials in which many of her patients participated. The combination and range of clinical and social needs are also too various for her to be guided by a simplified protocol. Like others, she relied on collaborative, multidisciplinary teams to support her judgement.

## EXTENDING PERSONALISING PATHWAYS

Protocols governing the organisation of trials and other studies intersect with clinical pathways but do not feature in them explicitly. For example, research participants may be followed up in a clinical research setting when they would otherwise have been referred back to primary care. The opening of a ‘trials clinic’ was one local solution we observed for patients, who wanted to be under the care of their oncologist, and for researchers, who wanted to collect longer term follow‐up data from these patients.

Faizia was an oncologist who I saw occasionally during the months I visited the hospital in the Hub or at staff meetings. She was one of thousands of healthcare workers who have trained in countries outside the United Kingdom but work in the NHS. When she moved to the United Kingdom, Faizia sought any work associated with oncology. For a time, she supported the hospital's open‐access follow‐up system, for which she assessed and stratified the needs of patients when their hospital care ended. After a year, a clinician leading the service invited her to take a renewable Specialty and Associate Specialist (SAS) contract in oncology to support a new clinic, managing the increasing number of patients who visit the hospital because they are part of a trial or study, monitoring and observing treatment over time. These patients may have been told that they are ‘cancer free’ and in partial or complete remission, but they were eligible to continue attending the hospital because they were research participants.

We met in relation to a research study called An Exploratory Breast Lead Interval Study (EBLIS) that we were following. EBLIS was an observational study funded by a large cancer charity and led by clinicians and other researchers working in an international collaboration (see Coombes et al., 2019). The study sought to track and establish novel biomarkers for the early detection of cancer relapse. If successful, this approach would lead to further translational research to validate and implement genetic monitoring, potentially changing clinical pathways in the future. Meanwhile, studies like EBLIS themselves transformed patient care because they involved visits to the hospital for blood monitoring every 6 months, integrating long‐term follow‐up and observation in a hybrid pathway that combined care and research. EBLIS was the first study to transfer patients from the busy patient lists of oncologists to what Faizia called her ‘trial clinic’, and she explained that, as drug and radiotherapy trials grew in number, this clinic grew in size.

Patients who attended Faizia’s trial clinic appreciated the opportunity to stay connected to clinical services. They saw their participation in research as a way to mitigate uncertainties connected to their previous cancer treatment, and to other non‐cancer health issues. Faizia as well as patients we interviewed noticed how they developed a rapport and what Faizia described as ‘continuity of care’. In contrast to the outpatients’ department where average waiting times remained 1.5–2 hours, participants in the trial clinic ‘get seen on time, they have enough time in the clinic with me’ to discuss concerns. In addition to the extra time and punctuality of the clinic, patients appreciated seeing specialists in oncology rather than general practitioners and expected research participation to speed up referrals back into oncology if needed. They valued their research participation in terms of the extended care they received.

Faizia also described limits to the care she can give. All the trials followed protocol, ‘you have to do the relevant examination, the blood tests, you’re always supported by the research team’. As a trained oncologist, she found her trial clinics ‘straightforward’ compared to the clinical work she did elsewhere in oncology. She had few difficulties, since she did not change or adapt treatment, and research participants did not require urgent or exceptional care, ‘they are so stable … they are just on follow‐up … I’m just assessing them in a general physical examination’. While she felt her expertise and experience as an oncologist was essential to the running of the trials clinic, she also felt her involvement in patient care was limited. She explained, ‘I’ve picked up a lot of recurrences as well in this clinic and the patients had to be called back to the oncology clinic.’ But she referred to other problems: ‘somebody has osteoarthritis of the knee, somebody has a heart problem, has mitral stenosis, needs a cardiology assessment, I don’t fiddle with that, that’s not my expertise …’ Administratively, research participants remained with the oncologists that treated them previously, and Faiza considered her involvement as a kind of triage, as she referred patients either to their named oncologists or to their GP for other referrals. But for research participants in studies like EBLIS these visits to her clinic were a solid link to the service, involving specialist expertise in oncology.

How patients experienced clinical pathways depended on their interactions with services over time. Some had lived with cancer for years and their evaluation of the service had an historical dimension. For example, after decades of local recurrences and many surgeries, Patricia attended a lecture at a voluntary, faith‐based organisation that encouraged her to get a genetic test, diagnosing her cancer as one driven by a BRCA1 mutation. At that time, this was not treatable and there were no trials in or through the hospital Trust for her to enter. She felt her genetic diagnosis and the cancer subtype were irrelevant to her treatment since there was no treatment appropriate to this cancer. When we met approximately 6 years after her BRCA1 diagnosis, she had entered a trial for a drug therapy for BRCA1 mutation carriers and she considered this treatment to be targeted to people with her genetic profile, but she did not see participating in the trial as a way of improving or extending her care. Like Sonia, Patricia saw changes in treatment as further proof that the medical team were organised in relation to cancers rather than their associated hosts. For Patricia, this focus on treatment came at the expense of other kinds of care that she valued: ‘I haven't been approached by anyone for a long time about me as a person, you know?’ she said, ‘I think that's a weakness in the system here’. Access to targeted, experimental therapies might be ‘just‐in‐time’ or even occupy the ‘right‐time’ from a biomedical perspective, but people evaluated this timeliness in the context of other care priorities.

When eligible, some patients found hope through participating in drug trials. Frances was diagnosed with metastatic disease of the liver and bone. She explained her shock and anxiety on learning that her cancer was already advanced, and she remembers being reassured by one oncologist at an early stage of her treatment—they said there were now hundreds of chemotherapies available to treat her. Not unlike Catherine's assessment that new treatments are added on a weekly basis or the senior oncologist who noted the speed at which treatments were being introduced, Frances felt her worries were managed in relation to the number of new drugs available to try. However, as her disease progressed and her liver tumours acquired resistance to different therapies, another doctor told her there were just two chemotherapies left to try. She was surprised at how her options had narrowed. When we met in the waiting area of the main oncology outpatients’ department, she described her plans to start the combination chemotherapy that her oncologist said would make her eligible for certain drug trials. She wanted to be eligible to extend her treatment options. In other words, she had begun to anticipate future research options that might extend her care. Subsequently, she sought genetic tests so as to understand more about the specificities of tumour evolution—she understood that the characteristics of her cancer were used to plot and plan her pathway. She was aware that a pathway could be formed in relation to possible alternatives, none of which guarantee better outcomes but would or might continue a course towards treatment. Her decision‐making began to anticipate a pathway that would emerge and change in relation to developments in her own and also others’ cancer and associated research.

## DISCUSSION

Patients and staff rarely referred to ‘personalisation’ or ‘personalised medicine’ in this hospital setting unless prompted to do so by our research group. Although adaptation, flexibility and revision are preconditions for what we term a ‘personalising clinical pathway’, the implementation of different features depends on uneven developments and interactions between care, research and data management activities. Slower, more uneven or delayed rates of implementation may be negatively labelled as ‘friction’ (see Bourret et al., 2021), particularly when compared to other expressions of ‘real time’ in other public and commercial services.

Developments we have described in ‘personalising cancer pathways’ have been built into clinical care at different times through a range of staff roles and systems, and their coordination remains challenging. These pathways required constant work, as we have described, as well as arduous evaluation against criteria of evidence, safety and care that are written into standards and enforced through professional and legal regulations. We have documented tensions, uncertainties and expectations that are generated for patients and staff adapting to an understanding of cancers, which is constantly updated (Cambrosio et al., 2018; Day et al., 2021). A more dynamic stratification that responds to cancer evolution affects how the service is managed, navigated and evaluated. Just as patients find their cancers defined and redefined using new techniques, clinical staff must update their knowledge and keep emerging evidence under constant review, leading them to maintain a balance between evidence‐based care and experimentation. Pathways develop iteratively through the treatment of individuals and groups associated with multiple schemes of classification (Lury & Day, 2019).

We ask about possible comparisons with algorithmic recommendation systems, for example, which also involve the tracking and repeated, recursive aggregation of data that, when disaggregated and applied, create novel pathways relating people, activities, preferences and purchases in marketing and advertising, retail, media, education, health, governance and security (see Amoore, 2020; Cheney‐Lippold, 2017; Lury & Day, 2019; Prey, 2018; Williamson, 2017). Rather than segmenting markets through existing sociodemographic categories, ‘personalising’ pathways in these environments, as with cancer medicine, aim to create relevant groupings of goods, services and users iteratively by aggregating and analysing data on their ‘journeys’ and uses. Though different in intention and outcome from one domain to another, interactions traced and tracked on media platforms are analysed with the aim of refining recommendations for products and services. The constant and extended approach to what counts as a relevant activity or behaviour to track means that infrastructures of service delivery involve a ‘steady accumulation of feedback loops, little circuits of interpretation and decision knit together into a vast textile’ (Seaver, 2018, p. 377).

Personalising pathways are not unique to medicine since other services are similarly concerned to reformat and refigure persons, products and populations more precisely into dynamic strata or segments. For instance, Mackenzie (2018) describes how algorithmic recommendation systems are used in online grocery shopping, while Seaver (2012, 2019) has described predictive ranking that blends what people like and who (or what) they resemble in media platforms using similar recommendation algorithms. Mackenzie and Seaver characterize these data‐driven systems as sociotechnical networks, crossing normative orders of personal and impersonal identity (Mackenzie, 2018, p. 13; Seaver, 2019).

Clinical pathways in cancer care also refine ‘recommendations’ to individual patients and groups of patients sharing a common condition based on dynamic combinations of comparative sociodemographic, diagnostic and treatment categories, and emergent groupings of ‘patients like you’. This is a logic of data‐driven comparison and preference that renders people or things ‘alike’ on a temporary basis, forming a key component to emergent practices of statistical reasoning (see Mackenzie, 2017). In this cancer service, we observed how interoperable data systems enable results from biological research, responses to treatment, digitised scans and integrated multiomics to inform and update diagnosis and treatment decisions rapidly in relation to ethical and legal regulations that shape responsibilities and duties of care. Data management informs the prescription of novel therapies and the management of research study data, as well as the capacities of buildings and staff to accommodate personalising pathways. Together, these shape pathways to a ‘real time’ that absorbs many unforeseen, contingent and historical waypoints in comparison with retail and social media platforms. Although personalising practices may share a common goal of establishing more precise and predictive tools to guide management and improve outcomes, personalised cancer medicine differs markedly in organisation and temporality from other practices in and beyond medicine. Algorithmic recommendation, for example, is used to create a near‐instantaneous tempo of automated likeness between internet users based on detailed behavioural data profiles that are then used to place advertisements and monitor conversion metrics (a system known as ‘real‐time bidding’ or RTB) (see Vurdubakis, 2019). Here financial profit or its promise—rather than the aggregated measure of life and quality of life—narrows definitions of what has or has not been ‘personalised’. Real time, at least in this context, has clearer (i.e. financialised) intervals that aim to measure whether or not online content has been ‘served’ and reached its ‘target’. Though online personalisation frequently promises ‘real time’ analysis (see Kant, 2020, p. 45, 159), according to the rapid delivery of targeted online content, the outcomes of personalised care pathways manufacture a very different (and differently distributed) ‘real time’. The process of embedding personalising practices of care and research, searching for what might be the ‘right’ intervention for the ‘right’ people at the ‘right’ moment, involves different materialities. The logic of adaptation and the integration of care and research activities within pathways has nonetheless transformed what counts as ‘real’ time for patients and staff. Our analysis has shown that one deferred outcome is the notion that a common pathway is known and predicted in advance, with all the difficulties and hopes that this time may hold for patients and staff. Patient journeys are increasingly extended with targeted interventions based on more frequent monitoring through a range of technologies, but predictable intervals that guided staff and patients along well‐defined and broadly shared clinical pathways also need to accommodate the uncertainties associated with learning heath systems.

## AUTHOR CONTRIBUTION

**William Viney:** Conceptualization (equal); Data curation (equal); Formal analysis (equal); Funding acquisition (supporting); Investigation (equal); Methodology (supporting); Project administration (equal); Writing – original draft (lead); Writing – review & editing (equal). **Sophie Day:** Conceptualization (lead); Data curation (equal); Formal analysis (equal); Funding acquisition (lead); Investigation (equal); Methodology (equal); Project administration (equal); Supervision (lead); Writing – original draft (equal); Writing – review & editing (equal). **Jane Bruton:** Formal analysis (supporting); Writing – review & editing (supporting). **Kelly Gleason:** Project administration (supporting); Resources (supporting); Writing – review & editing (supporting). **Charlotte Ion:** Project administration (supporting); Writing – review & editing (supporting). **Saima Nazir:** Writing – review & editing (supporting). **Helen Ward:** Conceptualization (supporting); Formal analysis (equal); Funding acquisition (lead); Investigation (equal); Methodology (equal); Supervision (supporting); Writing – review & editing (supporting).

## ENDNOTES

^1^
See https://pathways.nice.org.uk/ accessed 30 December 2020.

^2^
Here and elsewhere we use pseudonyms for individual staff members and patients.

^3^
These targeted anti‐cancer treatments act to arrest the cell cycle controlled by the D‐type cyclins and dependent kinases 4 and 6 (known collectively as CDK4/6).

^4^
Although not the focus of this research article, we also visited the laboratories and interviewed laboratory workers using these samples.
